# Wabash Valley Dental Society

**Published:** 1869-05

**Authors:** W. H. Pifer


### WABASH VALLEY DENTAL SOCIETY.

The annual meeting of the Wabash Valley Dental Association convened in the rooms of the Young Men's Christian Association, at Lafayette, Ind., Wednesday, the 24th day of March, 1869.

The following gentlemen were elected officers of the Association for the present year:

President—Dr. A. T. Keightley.

First Vice President—Dr. S. B. Brown.

Second Vice President—Dr. A. B. Cunningham.

Secretary—Dr. W. H. Pifer.

Treasurer—Dr. A. M. Moore.

Board of Sensors—Drs. A. M. Moore, A. B. Cunningham and S. B. Brown.

Executive Committee—Drs. E. V. Burt, J. S. Snoddy and S. H. Martin.

Delegates to American Dental Association—Drs. A. B. Cunningham, S. B. Brown, A. T. Keightley, W. H. Pifer, George D. Loag and S. H. Martin.

Contingents—Drs. A. M. Moore and J. S. Snoddy.

An address from the President elect met the approval of the members of the Association and was ordered for publication.

"When should the file be used?" was the title of an essay from Dr. W. F. Morrill, of New Albany, which was also requested for publication.

The Constitution and By-Laws of Indiana State Dental Association was adopted by the Association, after the necessary alterations.

The Code of Ethics, as adopted by the American Dental Association, was also adopted by the Association.

After an interesting and profitable meeting of two days, the Association adjourned, to meet at LaFayette, March 24, 1870.

The Wabash Valley Dental Association was organized in the spring of 1864, as a local Society, in the interests of the Dentists of Middle and Northern Indiana. It numbers from thirty-five to forty members. Meets annually at Lafayette. Though the minutes of many of the previous meetings have not appeared in the journals, the Society is in a flourishing condition.

W. H. PIFER, *Secretary.*

